# Improving the Antimicrobial Potency of Berberine for Endodontic Canal Irrigation Using Polymeric Nanoparticles

**DOI:** 10.3390/pharmaceutics16060786

**Published:** 2024-06-09

**Authors:** Célia Marques, Liliana Grenho, Maria Helena Fernandes, Sofia A. Costa Lima

**Affiliations:** 1 IUCS-CESPU, University Institute of Health Sciences (IUCS), Advanced Polytechnic and University Cooperative (CESPU), CRL, 4585-116 Gandra, Portugal; celia.m@netcabo.pt; 2 LAQV, REQUIMTE, Department of Chemical Sciences, Faculty of Pharmacy, University of Porto, Rua de Jorge Viterbo Ferreira, 228, 4050-313 Porto, Portugal; 3 BoneLab—Laboratory for Bone Metabolism and Regeneration, Faculty of Dental Medicine, University of Porto, 4200-393 Porto, Portugal; lgrenho@fmd.up.pt (L.G.); mhfernandes@fmd.up.pt (M.H.F.); 4 LAQV, REQUIMTE, ICBAS—School of Medicine and Biomedical Sciences, University of Porto, Rua de Jorge Viterbo Ferreira, 228, 4050-313 Porto, Portugal

**Keywords:** *Candida albicans*, endodontic irrigation, *Enterococcus faecalis*, gingival fibroblasts, PLGA nanoparticles

## Abstract

To address the challenges posed by biofilm presence and achieve a substantial reduction in bacterial load within root canals during endodontic treatment, various irrigants, including nanoparticle suspensions, have been recommended. Berberine (BBR), a natural alkaloid derived from various plants, has demonstrated potential applications in dentistry treatments due to its prominent antimicrobial, anti-inflammatory, and antioxidant properties. This study aimed to produce and characterize a novel polymeric nanoparticle of poly (lactic-co-glycolic acid) (PLGA) loaded with berberine and evaluate its antimicrobial activity against relevant endodontic pathogens, *Enterococcus faecalis*, and *Candida albicans*. Additionally, its cytocompatibility using gingival fibroblasts was assessed. The polymeric nanoparticle was prepared by the nanoprecipitation method. Physicochemical characterization revealed spheric nanoparticles around 140 nm with ca, −6 mV of surface charge, which was unaffected by the presence of BBR. The alkaloid was successfully incorporated at an encapsulation efficiency of 77% and the designed nanoparticles were stable upon 20 weeks of storage at 4 °C and 25 °C. Free BBR reduced planktonic growth at ≥125 μg/mL. Upon incorporation into PLGA nanoparticles, 20 μg/mL of [BBR]-loaded nanoparticles lead to a significant reduction, after 1 h of contact, of both planktonic bacteria and yeast. Sessile cells within biofilms were also considered. At 30 and 40 μg/mL, [BBR]-loaded PLGA nanoparticles reduced the viability of the sessile endodontic bacteria, upon 24 h of exposure. The cytotoxicity of BBR-loaded nanoparticles to oral fibroblasts was negligible. The novel berberine-loaded polymeric nanoparticles hold potential as a promising supplementary approach in the treatment of endodontic infections.

## 1. Introduction

The objective of endodontic treatment is to achieve biomechanical cleaning of an infected root canal system, eliminating intracanal pathogens and infected tissue, preventing further infection, and promoting healing of apical periodontitis [1]. However, clinical studies have shown that the treatment may fail if microbial biofilms persist in the root canal system [2]. Antibiotic resistance of the microorganisms and the complex anatomy of the canal system, particularly in the root apical third, may contribute to this drawback. Additionally, microorganisms typically present themselves in complex biofilms, which can prevent the efficacy of the irrigating solutions during the irrigation procedure [3,4]. It is recommended to use irrigants with a broad antimicrobial spectrum and efficacy on biofilms that are not caustic or cytotoxic to periapical tissues [5,6,7,8].

Sodium hypochlorite (NaOCl) has been widely used as an intracanal irrigant for its strong antimicrobial and tissue-dissolving properties. It can eliminate necrotic tissue and vital pulp substrates, but it cannot remove inorganic components of the smear layer. Studies have shown that it is more effective against biofilms at concentrations of 6% (*w/v*) compared to 1 and 2% (*w/v*) [4,9]. However, a strong correlation exists between the concentration of NaOCl and the increased risk of adverse effects for the patient when accidentally contacting the periradicular tissues. Additionally, NaOCl has negative effects on the mechanical properties of dentin, such as hardness, roughness, modulus of elasticity and flexural strength [5,6,10].

Chlorhexidine (CHX) is an irrigating solution used in endodontics. It has the unique property of substantivity but cannot dissolve organic and inorganic matter, therefore it cannot be used as a single irrigating solution. CHX’s antimicrobial efficacy is due to its action on the microorganism’s cell wall, leading to its death [7]. Wang and co-authors found that 6% (*w/v*) NaOCl has better antibiofilm performance than 2% (*w/v*) CHX [11].

Recent studies indicate that there is no significant difference in the antibacterial effectiveness of NaOCl and CHX against *Enterococcus faecalis* (*E. faecalis*), *Staphylococcus aureus*, *Streptococcus salivarius*, and *Actinomyces israelii*. However, it is important to note that some studies did not report clinically relevant outcomes such as the total volume of irrigant used and/or the exposure time [12]. It is crucial to consider these limitations when interpreting the results. 

There is a need for new agents with antimicrobial activities that are safe for human use, thereby minimizing the risks to the surrounding tissues and the patients. Natural compounds obtained from plants have attracted attention due to their potential biological activities, easy accessibility and as a promising solution to combat mechanisms of pathogen resistance [13]. Berberine (BBR) is an isoquinoline alkaloid that is present in the roots, rhizome, and stem bark of some plants of the *Berberidaceae* family [14]. The potential pharmacological and therapeutic relevance of BBR has been suggested for a variety of conditions [14,15]. Among them, promising antidiabetic effects have been described [16,17] and this drug was tested in a few clinical trials. The information is further complemented in studies with animal models and cell culture methodologies to disclose the involved mechanisms [16,18,19]. The antidiabetic effect appears to be ascribed to several mechanisms [18,19] including its anti-inflammatory and antioxidant activities [20]. Other potential effects include protection against neurodegenerative diseases [21,22] as well as anticancer [23], antiviral [24], antifungal [25,26], and antibacterial activity [27,28]. For most of these effects, significant available information arose from in vivo and in vitro studies and further work is needed in order to substantiate the therapeutic security [28]. 

BBR is classified as a class IV drug in the Biopharmaceutical Classification System due to its low aqueous solubility of approximately 2.0 mg/mL. As a result, it has low oral bioavailability, further worsened by its self-aggregation in the gastro-intestinal track decreasing its absorption. This limitation requires higher dosages to achieve the desired effects which may compromise patient safety and increase adverse effects [29,30]. The reported safety dosage for systemic effects following oral administration is dose- and time-dependent and varies widely [29]. On the other hand, the pharmacological relevance of BBR associated to topical/local use is suggested in a variety of studies, namely concerning its established antimicrobial effects for a range of microorganisms [28]. In an endodontic context, the effect of BBR has been focused on antibacterial pathogens, the main microbial agents present in the root canal system and in persistent endodontic infections [31,32]. Among them, *E. faecalis* plays an essential role in endodontic lesions after root canal treatment, being found in a high percentage of treated teeth with apical periodontitis, as a single organism or as part of bacterial flora [33]. It penetrates into the dentinal tubules and its removal during root canal therapy is very difficult. Additionally, it tolerates adverse environmental conditions and has the ability to form biofilms, explaining its high prevalence [33]. On the other hand, *Candida albicans* (*C. albicans*) is the fungus most frequently isolated from endodontic root canal infections [34]. Within biofilms it is very resistant to antifungal treatment, because the cell growth and metabolism are slowed and protected by extracellular polymeric substances [34]. Given their documented relevance for endodontic infections, *E. faecalis* and *C. albicans* were selected for this study.

In recent decades, there has been a growing interest in the physicochemical properties of nanosized delivery systems, particularly in improving bioavailability of poor water-soluble molecules [35,36,37]. A recent comprehensive review explored various nano strategies developed for delivering BBR, highlighting the advantages of employing nanotechnology for its effective administration [38]. The use of a nano-based irrigant solution could enable the local delivery of a high concentration of the antimicrobial agents while preventing adverse side effects [39,40]. Various types of nanoparticles have been studied for their effectiveness in endodontic disinfection [41]. Among the organic nanoparticles, polymeric nanoparticles such as chitosan and poly lactic-co-glycolic acid (PLGA) nanoparticles are particularly relevant [41,42]. PLGA is a synthetic biodegradable polymer favored in drug delivery for its biocompatibility, ease of use, and tunable nature. PLGA offers a versatile platform for oral drug delivery, making it a preferred choice for producing nanoparticles targeted for applications in the oral cavity [43]. Regarding the inorganic ones, metallic NPs such as silver, gold, iron, copper and zinc have received the most attention [44]. While these nanoparticles demonstrate the capacity to produce reactive oxygen species including superoxide, hydrogen peroxide and hydroxyl radicals, inducing oxidative cellular damage to bacteria [45], their drawbacks lie in toxicity [46].

The current study aims to introduce a novel approach using polymeric nanoparticles for the first time as a delivery tool for BBR in the context of endodontic irrigation. The designed nanoparticles were characterized regarding particle size, surface potential, morphology, BBR content, chemical interactions, in vitro drug release and storage stability. Furthermore, the antioxidant activity and the biological profile concerning antimicrobial activity against *E. faecalis* and *C. albicans*, and the toxicity towards human gingival fibroblasts, were also assessed.

## 2. Materials and Methods

### 2.1. Materials

Berberine chloride hydrate ≥ 90%; poly (vinyl alcohol) (PVA) 87–90% hydrolyzed; Dulbecco’s phosphate-buffered saline (DPBS) (10×); 2,2-azinobis-(3-ethylbenzothiazoline-6-sulfonic acid) radical cation decolorization test (ABTS assay); 2,2-diphenyl-1-picrylhydrazyl radical (DPPH); and methanol were supplied from Sigma-Aldrich (St Louis, MO, USA). Poly (lactic-coglycolic acid) (PLGA) (50:50 Purasorb^®^ PDLG 5004A) was obtained from Purac Biomaterials (Amsterdam, the Netherlands). Acetone and ethanol, absolute, >= 99.8% from Fisher Scientific (Bishop Meadow Road, Loughborough, UK). Double-deionized water was provided by an ultra-pure water system (Arium Pro, Sartorius AG, Göttingen, Germany). For cell culture studies, the following suppliers were used: human gingival fibroblasts (AG09319, Coriell Institute, Camden, NJ, USA), alpha-minimum essential medium (α-MEM), fetal bovine serum (FBS), penicillin, streptomycin and amphotericin B (Gibco, Waltham, MA, USA), calcein-AM (BioLegend, San Diego, CA, USA), propidium iodide (PI, BD Biosciences, Franklin Lakes, NJ, USA), dimethyl sulfoxide (DMSO) (Panreac, Darmstadt, Germany), 3-(4,5-Dimethylthiazol-2-yl)-2,5-Diphenyltetrazolium Bromide (MTT) (Sigma-Aldrich, St. Louis, MO, USA), formaldehyde (Sigma-Aldrich, St. Louis, MO, USA), Alexa Fluor-conjugated phalloidin (Alexa Fluor^®^ 488 Phalloidin, BioLegend, San Diego, CA, USA) and Hoechst 33342 (BioLegend, San Diego, CA, USA). For the antimicrobial evaluation, the used reference strains were *Enterococcus faecalis* (ATCC 29212) and *Candida albicans* (ATCC 10231). Tryptic Soy Broth (TSB) and Sabouraud Dextrose Broth (SDB) were acquired from Liofilchem (Roseto degli Abruzzi (TE), Italy).

### 2.2. Preparation of Berberine-Loaded PLGA Nanoparticles

BBR-loaded PLGA nanoparticles were prepared by nanoprecipitation method followed by sonication at room temperature [47]. The preparation process involved mixing the organic phase composed of acetone (10 mL) with PLGA (10 mg) and then with the aqueous phase consisting of 1% (*w/v*) PVA (20 mL). After the self-assembly, the formulation was subjected to sonication, using a probe sonicator (VCX130, Sonics and Material Vibra-Cell TM with a CV-18 probe; 115 Newtown, Westport, CT, USA) at 70% of amplitude for 1 min. To obtain BBR-loaded nanoparticles, 1 mg of the bioactive compound was previously dissolved in 1 mL of ethanol (absolute) using an ultrasound bath and added into the organic phase prior the sonication step, promoting its inclusion in the nanoparticle matrix. After solvent evaporation overnight, at room temperature on a magnetic stirring plate (IKA-Werke, Staufen, Germany), the nanoparticles were recovered.

### 2.3. Characterization of Berberine-Loaded Nanoparticles

#### 2.3.1. Size, Polydispersity Index and Zeta Potential

The mean size, polydispersity index (PDI), and zeta potential of the nanoparticles in aqueous solution pH 6.5 were determined by dynamic light scattering (DLS) using a ZetaPALS, Zeta Potential Analyzer (Brookhaven Instruments Corporation; Holtsville, NY, USA). For each assay, size measurements were carried out at room temperature in 6 runs of 2 min. The zeta potential of the nanoparticles was determined using an electrode operating at a scattering angle of 90°. Six runs of 10 cycles at room temperature for each assay were performed. The obtained values are the average values from the 6 runs. 

#### 2.3.2. Encapsulation Efficiency and Drug Loading Determination

The quantification of BBR incorporated within the PLGA nanoparticles was determined indirectly by measuring the non-entrapped compound using ultra-violet (UV-Vis) spectrophotometry. Briefly, the supernatant was collected after 1 mL nanoparticles centrifugation at 10,000× *g*, 20 °C for 30 min, using an Allegra X-15R centrifuge (Beckman Coulter, Brea, CA, USA). The amount of BBR in the supernatant was quantified by an UV-Vis spectrophotometer (Jasco V-660 Spectrophotometer, Software: Spectra Manager v.2. Jasco Corporation, Easton, MD, USA) at 350 nm. A calibration curve of berberine in ethanol was established from 0.005 to 0.09 mg/mL. The entrapment efficiency (EE) and the drug loading (DL) were calculated using the following equations:

EE (%) = (Initially added BBR-Non-entrapped BBR)/(Initial added amount of BBR) × 100

DL (%) = (Entrapped drug)/(Initial polymer mass + initial BBR mass) × 100

#### 2.3.3. Morphology Assessment

Nanoparticles morphology was observed by transmission electron microscopy (TEM, Jeol JEM-1400, Tokyo, Japan). Images were obtained after one drop of nanoparticles suspension was placed over a grid followed by negative staining with uranyl acetate and placed at the accelerating voltage of 60 kV. 

#### 2.3.4. Storage Stability

To evaluate the nanoparticles stability over a storage period, freshly prepared nanoparticles were stored in glass vials at 4 °C and room temperature, protected from light. The characterization involved determination of size, PDI, zeta potential and drug content periodically up to 5 months, as previously described.

#### 2.3.5. Fourier Transform Infrared Spectroscopy

Samples of unloaded and BBR-loaded PLGA nanoparticles were freeze-dried. After overnight freezing at −80 °C (Deep freezer, GFL^®^, Burgwedel, Germany), the samples were left for 72 h in a LyoQuest 85 plus v.407 Telstar freeze dryer (Telstar^®^ Life Science Solutions, Terrassa, Spain). Lyophilized samples of nanoparticles, and commercial powders of BBR and PLGA were evaluated in a Fourier transform infrared spectroscopy (FTIR). Spectra were collected by a PerkinElmer^®^ Spectrum 400 (Waltham, MA, USA) equipped with an attenuated total reflectance (ATR) device and zinc selenite crystals. Samples were transferred directly to the ATR device, compressed, and the results were obtained with a combination of 16 scans. The spectra and the resolution range were 4000–600 cm^−1^ with a resolution of 4 cm^−1^.

#### 2.3.6. *In Vitro* Drug Release Assay

The in vitro release of BBR from BBR-loaded PLGA nanoparticles was performed to analyze the drug release under mimetic physiological conditions. Sink conditions are crucial to ensure drug release evaluation. It ensures that the release process is predominantly governed by the intrinsic dissolution properties of the compound rather than being limited by its solubility in the medium. Given that BBR water solubility is around 2 mg/mL [48], the assay was carried out in sealed tubes containing 100 μg of BBR in a volume of 1 mL phosphate buffer pH 7.4, shacked in an orbital stirrer (KS 3000 IC IKA-Werke, Staufen, Germany) set at 180 rpm and 37 °C. At regular intervals, 0, 2, 4, 24, 48 and 120 h, a tube containing 1 mL sample was centrifuged at 10,000× *g*, 20 °C for 30 min using an Allegra X-15R centrifuge (Beckman Coulter, Brea, CA, USA). The supernatant was recovered for BBR quantification. The content of BBR was determined by UV-Vis spectrophotometer as described above. Several mathematical models for evaluation of drug release kinetics (zero order, first order, Higuchi, Peppas-Korsmeyer and Hixon-Crowell) were fitted to the experimental data [49]. The kinetic model exhibiting the highest regression coefficient (r^2^) was determined to be the mechanism by which release occurred.

#### 2.3.7. Antioxidant Activity

The ABTS and the DPPH assays were performed to assess the antioxidant activity of BBR as free and as loaded within the nanoparticles. Briefly, an ABTS solution was prepared by adding an equal volume of 7 mM ABTS and of 2.45 mM potassium persulfate solution in a flask. Upon an overnight period protected from light, at room temperature, the ABTS solution was diluted in water to achieve an absorbance of 0.90 ± 0.02 at 734 nm. The assay was then performed using a 96-well plate, mixing 50 µL of ABTS with 50 µL of each sample and negative control (only ABTS). After 30 min of incubation at 25 °C, protected from light, the absorbance was determined in a plate reader Synergy^TM^ HT Multimode plate reader (BioTek^®^ Instruments Inc., Winooski, VT, USA). For the DPPH assay, a working solution was prepared with 3.94 mg of DPPH in 50 mL cold methanol. To a 96-well plate, 100 µL of the DPPH solution was added to 100 µL of the BBR-free solution and loaded in PLGA nanoparticles (10, 15, 20 and 25 μg/mL in BBR). After 30 min of the reaction in the dark, the absorbance was read at 515 nm. In each assay, ethanol (100 µL) plus water (100 µL) was used as a blank sample (Abs_blank_). DPPH (100 µL) plus methanol (100 µL) was a negative control sample (Abs_control_), and all measurements were carried out in triplicate [50]. The percentage antioxidant activity (AA%) was calculated according to the following equation:

AA% = 100 − [(Abs_sample_ − Abs_blank_) × 100]/Abs_control_

### 2.4. Antimicrobial Activity of Berberine-Loaded PLGA Nanoparticles

#### 2.4.1. Microorganisms and Culture Conditions

*Enterococcus faecalis* ATCC 29212 and *Candida albicans* ATCC 10231 were used as reference strains. Prior to each experiment, *E. faecalis* was cultured in TSB, while *C. albicans* was cultured in SDB, both for 24 h at 37 °C and 120 rpm. Following incubation, the initial suspensions used in the subsequent assays were prepared from microorganisms in the exponential growth phase, within their respective broth media, by measuring their optical density at 600 nm.

#### 2.4.2. Antimicrobial Activity of Berberine-Loaded PLGA Nanoparticles

*Planktonic population*. Suspensions of both *E. faecalis* and *C. albicans* were prepared to reach a final concentration of 10^6^ colony-forming units (CFUs)/mL. These microbial suspensions were exposed, for 1 h, to PLGA nanoparticles, loaded with or without BBR, at final concentrations of 10, 15, 20, 25 and 40 μg/mL in BBR equivalent to 0.140, 0.215, 0.285, 0.35, and 0.57 mg/mL in polymer. Control cultures were prepared in a similar way, excluding the addition of PLGA nanoparticles. After incubation, the supernatants were serially diluted and plated onto the appropriate agar plates. After a 24 h incubation period at 37 °C, CFUs were counted and the results were plotted as a percentage of the control, set at 100%. 

*Sessile population*. The effect of BBR-loaded PLGA nanoparticles on biofilm formation was tested using a static biofilm formation assay. Suspensions of *E. faecalis* and *C. albicans*, at 10^8^ CFU/mL, were inoculated into 96-well plates and incubated for 4 h at 37 °C to allow microbial adhesion. Subsequently, the supernatants were removed, and the wells were carefully rinsed with 0.9% NaCl to remove planktonic and weakly adhered cells. The adhered microorganisms were then exposed to PLGA nanoparticles, loaded with solutions at concentrations of 20, 30 and 40 µg/mL, followed by a 24 h incubation at 37 °C and 120 rpm. Empty PLGA nanoparticles were added at equivalent concentrations of 0.285, 0.430, and 0.570 mg/mL. Control cultures were prepared in a similar way, excluding the addition of PLGA nanoparticles. After incubation, sessile cells were quantified using CFUs. Briefly, each well was rinsed twice and the bottom of the wells, containing 100 µL of 0.9% NaCl, was scraped to detach the sessile microorganisms. The resulting suspensions were diluted and plated onto the appropriate agar plates. After 24 h at 37 °C, CFUs were counted and the results were plotted as a percentage of the control, set at 100%. 

*Antimicrobial activity of berberine*. For comparison, the antimicrobial activity of free berberine was assessed also against planktonic and sessile *E. faecalis* and *C. albicans*, using the experimental setup described above for the testing of the BBR-loaded nanoparticles. BBR was tested at final concentrations of 125, 250 and 500 μg/mL, which was the concentration range that exhibited antimicrobial activity. 

### 2.5. Cytocompatibility of Berberine-Loaded PLGA Nanoparticles

The cellular toxicity of BBR-loaded PLGA nanoparticles and unloaded nanoparticles was evaluated against human gingival fibroblasts. Cells were seeded into 96-well plates, at a density of 10^4^ cells/cm^2^ and incubated for 24 h. After cell adhesion, the medium was removed, and cells were exposed to different concentrations of BBR-loaded PLGA nanoparticles and unloaded nanoparticles (0 to 25 µg/mL in BBR). The plates were incubated for 1 h. The viability of the cell layer was assessed by the live/dead assay and the MTT reduction assay. Morphology and F-actin cytoskeleton organization were analyzed in immunostained cultures. Untreated cells were taken as negative control with 100% viability (absence of toxicity). 

*Live/dead assay*. After 1 h exposure to the nanoparticles, cultures were washed with PBS and stained with calcein-AM (1 µM) to visualize live cells, and with propidium iodide (50 µL/mL) to detect dead cells. Cultures’ fluorescence was imaged by a Celena S digital imaging system (Logos Biosystems, Anyang-si, Korea), with live cells staining green and dead cells staining red. *MTT assay*. A working solution of MTT was prepared in complete culture medium at 5 mg/mL. After the period of incubation, the supernatant of the cell cultures was removed and the MTT solution added and left for 3 h at 37 °C. Viable cells, through the mitochondrial activity, can reduce the MTT compound resulting in formazan violet-blue crystals. Prior determination of the absorbance, at 550 nm, was carried out in a microplate reader (Synergy HT, Biotek, VT, USA), and the crystals were dissolved in DMSO for a duration of 15 min. *F-actin cytoskeleton*. The cellular morphology and cytoskeletal organization were evaluated in control and treated cultures. For the immunostaining of F-actin cytoskeleton and nuclei, cells were fixed (3.7% formaldehyde, Sigma-Aldrich; 10 min), washed twice with PBS, and stained for F-actin with Alexa Fluor- conjugated phalloidin diluted 1:100 in BSA/PBS (1%; 30 min). Samples were washed with PBS and the nuclei counterstained with Hoechst 33342 (8 μg/mL) for 15 min. Cells were photo documented by a Celena S digital imaging system.

### 2.6. Statistical Analysis

Biological assays were conducted with three biological replications, each one with three technical replicas. One-way analysis of variance (ANOVA) and Tukey post hoc test were used to determine the differences between the multiple groups. Data are expressed as mean ± SD, and *p*-values < 0.05 were considered statistically significant.

## 3. Results and Discussion

### 3.1. Characterization of Polymeric Nanoparticles Containing Berberine

The PLGA nanoparticles were obtained by the nanoprecipitation method in the presence of a surfactant (PVA). The main physicochemical characteristics of the PLGA nanoparticles are listed in Table 1.

The PLGA nanoparticles and BBR-loaded PLGA nanoparticles were characterized by mean hydrodynamic diameter, polydispersity index, zeta-potential, and encapsulation efficiency, as shown in Table 1. The diameter of PLGA and BBR-loaded PLGA nanoparticles was found to be, respectively, 148 ± 2 and 140 ± 2 nm, suggesting that the incorporation of BBR did not influence the formulation’s diameter. BBR has been previously loaded into polymer–lipid hybrid nanoparticles resulting in a mean size of 150 ± 5 nm [51]. The developed nanoformulations are monodisperse, presenting a PDI of approximately 0.25 (Table 1). PDI values bellow 0.3 denote the presence of two to three different populations with a slender size distribution [52]. The surface potential obtained ranged between −6 ± 1 to −4 ± 1 mV for PLGA and BBR-loaded PLGA nanoparticles. The surface characteristics of PLGA/PVA nanoparticles result from the absorption of PVA, a polymer composed of partially hydrolyzed poly(vinyl acetate) with vinyl alcohol and vinyl acetate monomer units. This led to its incorporation within the PLGA matrix and on the surface of the nanoparticles. Given PVA’s considerable molecular weight (30–70 kDa), its layer effectively shielded the PLGA charges, resulting in an almost neutral zeta potential value [53]. Of notice, the encapsulation of BBR did not affect the surface charge of the polymeric nanoparticles. The EE values in BBR-loaded PLGA nanoparticles were approximately 70%, equivalent to 700 μg of loaded berberine. This result is quite promising as others using lipid nanoparticles obtained an encapsulation efficiency of only 36% [51]. The drug loading capacity obtained for BBR-loaded PLGA nanoparticles was around 7%. This loading capacity indicates the maximum amount of drug encapsulated efficiently within the nanoparticle structure. Greater drug loading capacities typically lead to more efficient drug delivery systems, possibly necessitating lower doses for therapeutic benefits.

**Figure 1:**
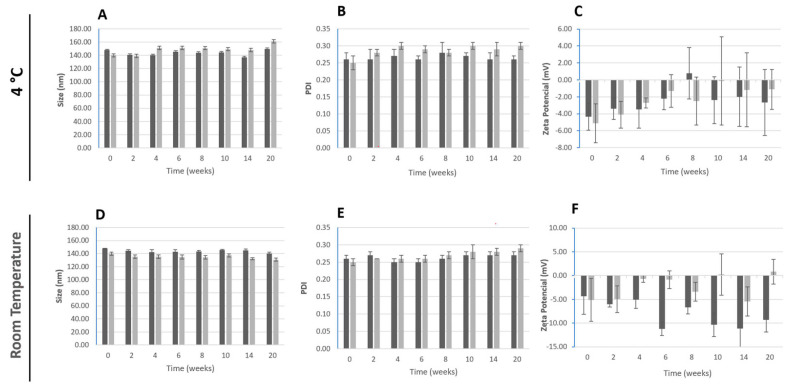
Storage stability of PLGA nanoparticles at 4 °C and at room temperature (25 °C). Parameters of size (**A**,**D**), PDI (graphs **B**,**E**) and zeta potential (**C**,**F**) were determined for BBR-loaded PLGA (black), and empty PLGA nanoparticles (light grey). Each result represents the mean ± standard deviation for n = 6 measurements.

The surface charges obtained for the developed PLGA nanoparticles are low and might not assure enough electrostatic repulsions to avoid the nanoparticles aggregation [54]. Therefore, the storage stability of the unloaded and BBR-loaded (Figure 1) PLGA nanoparticles was studied over 20 weeks. Physical-chemical stability was assessed by analyzing the parameters: particle size, PDI, zeta potential and drug content of the polymeric nanoparticles in relation to freshly prepared nanoparticles, when stored at 4 °C and at room temperature (25 °C and protected from light). According to the obtained results, no significant alterations were detected in relation to the freshly obtained BBR-loaded nanoparticles for both storage conditions, as size values were between 130 and 160 nm (Figure 1), and the PDI was below 0.3. Despite the observed variability over the period of study, the surface charge remained around—5 mV (Figure 1). The presence of BBR did not influence these physicochemical properties of the PLGA nanoparticles. The PLGA nanoparticles were able to retain 95% of BBR for the 20 weeks, at both storage conditions.

The morphology of the PLGA nanoparticles was evaluated by TEM (Figure S1). The spheric nanoparticles present a bright core, and the presence of BBR did not influence the morphological features of the polymeric nanoparticles.

To evaluate possible chemical interactions between the BBR and the PLGA nanoparticles, FTIR spectroscopy was conducted (Figure S1). The spectra of both PLGA and BBR-loaded PLGA nanoparticles showed a broader characteristic peak of PLGA at 1750 cm^−1^ (carbonyl C=O stretching band) which illustrates the presence of an ester group of PLGA [55]. The BBR IR characteristic peaks are identified as 1103 cm^−1^ (C-O), 1597 cm^−1^ and 1504 cm^−1^ (aromatic C=C stretching), 1504 cm^−1^ (skeleton vibration of aromatic C=C ring stretching), 1387 cm^−1^ and 1362 cm^−1^ (C=C stretching), 1277 cm^−1^ (C-O-C stretching), and 1036–1184 cm^–1^ (in plane = C-H bending) [56]. Due to the greater quantity of PLGA compared to BBR within the nanoparticle, the peaks associated with the polymer reduce those of BBR.

### 3.2. Evaluation of the In Vitro Berberine Release

**Figure 2:**
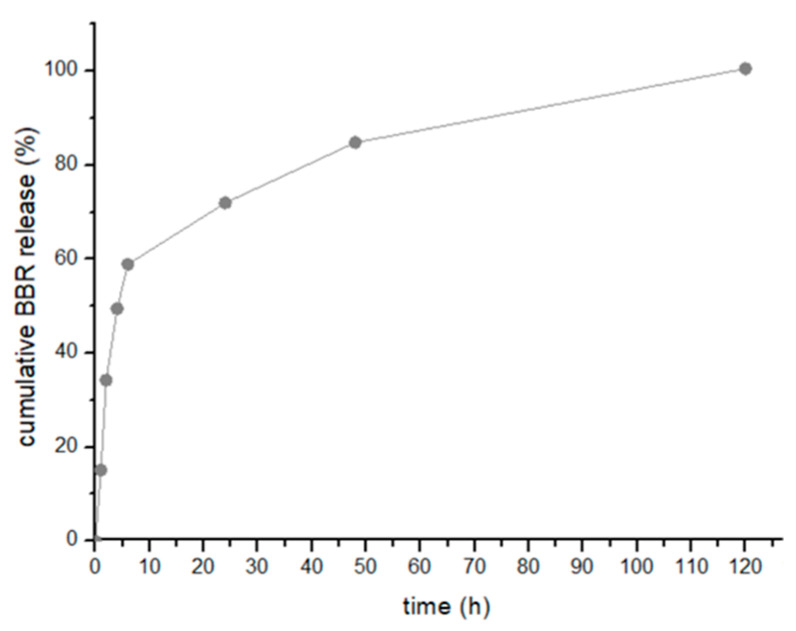
In vitro release of BBR from PLGA nanoparticles. Cumulative BBR release of BBR-loaded PLGA nanoparticles determined at physiological pH. Data are shown as mean ± standard deviation from n = 3.

BBR demonstrated a sustained release pattern from the PLGA nanoparticles under in vitro conditions (Figure 2).

The BBR release was studied at physiological conditions (37 °C, pH 7.4) and revealed a sequential and controlled release. Figure 2 shows that the PLGA nanoparticles released 97 ± 3% of BBR of the total entrapped BBR within 5 days. BBR showed an initial fast release (8–35%) followed by a sustained pattern until 97% was released after 120 h. The release rate of BBR from the polymeric nanoparticles is slow, over 5 days, thus confirming the controlled drug release from the PLGA core. The drug release mechanisms from polymeric nanoparticles depends on the properties of the drug delivery system, and the characteristics of the drug. The results obtained in this study led to infer that the drug release was governed by diffusion of the drug from the polymeric core structure, as described by the Higuchi mathematical model, r^2^ of 0.9906 (Table S1). Indeed, within polymeric nanoparticles, the drug is evenly distributed throughout the matrix. Consequently, the release takes place through drug diffusion, and both surface and bulk erosion of the matrix [57]. These processes led to the slow drug release. Polymeric nanoparticles are characterized to release cargo primarily governed by Fickian diffusion of the drug molecules through the matrix [58]. This process results in controlled drug release.

### 3.3. Berberine Antioxidant Activity

Berberine has been extensively studied for its antioxidant effect. Hence, in order to understand how its encapsulation in the PLGA nanoparticles affects BBR’s antioxidant capacity, ABTS and DPPH assays were performed. These two distinct antioxidant assays are representative of different mechanisms, such as hydrogen atom transfer or/and single electron transfer mechanism, and reaction conditions, such as pH, time, and temperature [59]. The ABTS reaction is based on a mechanism involving hydrogen atom transfer or/and single electron transfer mechanism, allowing the quantification of the quenched ABTS•+ radical cation, within a broad pH range and in both hydrophilic and lipophilic samples [60]. On the contrary, the DPPH assay depends only on a single electron transfer mechanism, whereas the DPPH• is scavenged, a process influenced by the solvent and the pH [61]. This method depends on proton transfer to reduce the radical and is performed in an organic solvent, methanol, that may bring some solubility resulting in a low activity of the bioactive compound.

**Figure 3:**
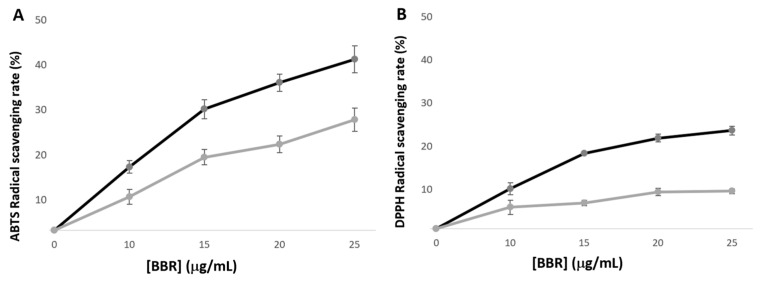
Antioxidant activity of BBR-loaded nanoparticles. (**A**) ABTS and (**B**) DPPH radical scavenging activity percentage of free BBR (black line) and BBR-loaded PLGA nanoparticles (grey line). Values correspond to means ± standard deviation for n = 3 replicates.

Figure 3 shows that the scavenging activity of BBR-loaded nanoparticles as well as free BBR is concentration dependent. Furthermore, incorporation of BBR within polymeric nanoparticles decreases the antioxidant activity of BBR compared to its free form. The ABTS assay is governed by two mechanisms leading to higher activity and faster kinetics in relation to DPPH. Additionally, since DPPH is conducted in an organic solvent, methanol, it may pose solubility concerns, leading to decreased activity of the extract. In fact, the nanoparticle’s polymeric core is not soluble in methanol, limiting the BBR available for the DPPH scavenging activity. Albumin or alginate-chitosan based nanoparticles have been described to exhibit enhanced BBR’ DPPH activity, most probably related to the nanoparticle’s core solubility [61,62]. Nevertheless, the BBR loaded into PLGA nanoparticles still exerts DPPH scavenging activity. In this study, only the BBR released from PLGA nanoparticles is assessable in the antioxidant assays. Due to the gradual release of BBR from the nanoparticles in the first hours, the antioxidant activity observed is lower compared to free BBR in solution (Figure 3).

### 3.4. Antimicrobial Activity

During root canal disinfection, the endodontic irrigant is applied for a short time but its antimicrobial activity would be expected to target both planktonic and sessile microorganisms still present in the root canal structure following initial instrumentation. Berberine has been reported to exhibit antimicrobial activity against a range of microorganisms, including two major endodontic pathogens, *E. faecalis* and *C. albicans* [33]. A serious drawback of BBR is its poor solubility, which hampers its movement through biological structures, implying the use of high levels to achieve active concentrations to produce the desired effects. As such, tissue and cellular toxicity associated with active BBR levels is often described, being an important limiting factor to its therapeutic use [28,29]. Considering this, in the present study, PLGA nanoparticles were loaded with BBR aiming to produce easily diffusible entities to allow the targeted delivery of BBR overcoming the need to use high doses. In this context, BBR as a free molecule and PLGA-loaded BBR nanoparticles were compared in terms of their efficacy against two endodontic pathogens, the Gram (+) *E. faecalis* and the yeast *C. albicans*. 

**Figure 4:**
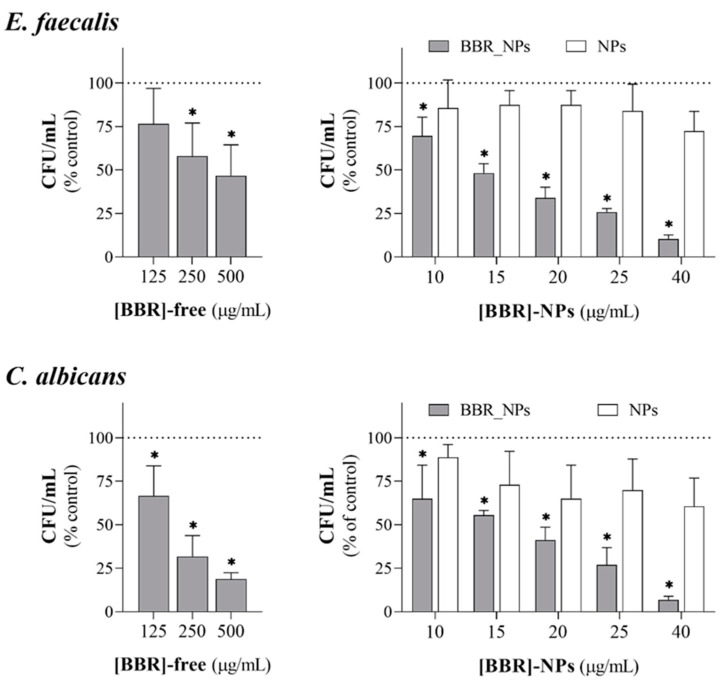
Antimicrobial activity of the BBR-free drug ([BBR]-free) and BBR-loaded PLGA nanoparticles ([BBR]-NPs) on planktonic *E. faecalis* and *C. albicans* growth, for the concentration range of 125 to 500 µg/mL ([BBR]-free) and 10 to 40 µg/mL ([BBR]-NPs), and 1 h exposure time. Unloaded PLGA NPs (NPs) were also assessed. * *p* < 0.05, denotes significant differences compared to the control (set at 100%, dotted line).

Figure 4 summarizes the results for the inhibitory concentration range of BBR-free drug and BBR-loaded PLGA nanoparticles against planktonic *E. faecalis* and *C. albicans*. Exposure time was setup to 1 h, taking into consideration that within the short clinical timeframe of the endodontic irrigation, the planktonic microbial population would be the most affected. In these experimental conditions, the BBR-free drug displayed inhibitory effects at concentrations similar and higher than 125 µg/mL against both pathogens, following a distinctly concentration-dependent pattern. *C. albicans* displayed slightly greater susceptibility to the BBR-free drug, i.e., at 500 µg/mL; the inhibitory effect was ~50% for *E. faecalis* and ~80% for *C. albicans*. On the other hand, BBR-loaded PLGA nanoparticles exhibited similar dose-dependent inhibitory effects in significantly lower concentrations, corresponding to final concentrations of 10 to 40 µg/mL in BBR. As such, planktonic *E. faecalis* and *C. albicans* were highly susceptible to BBR-loaded nanoparticles. BBR is reported to act via multiple and dose-dependent mechanisms encompassing intracellular and surface effects targeting specific and/or nonspecific entities or events [63]. The high activity in the planktonic pathogens might be associated with an easy cellular diffusion of the loaded nanoparticles with subsequent BBR release accomplishing inhibitory effects in microbial intracellular key mechanisms. Additionally, it was previously shown that BBR within the PLGA nanoparticles exerts antioxidant activity (Figure 3), also contributing to the effect observed in the studied endodontic pathogens.

**Figure 5:**
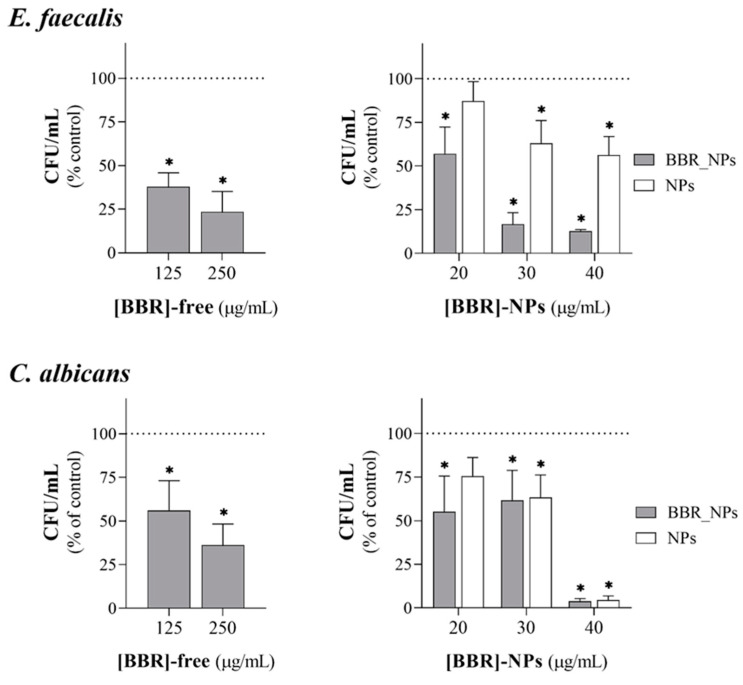
Antimicrobial activity of the BBR-free drug ([BBR]-free) and BBR-loaded PLGA nanoparticles ([BBR]-NPs) on sessile *E. faecalis* and *C. albicans* growth, for 125 and 250 µg/mL ([BBR]-free) and 10 to 40 µg/mL ([BBR]-NPs), and 24 h exposure time. Unloaded PLGA NPs (NPs) were also assessed. * *p* < 0.05, denotes significant differences compared to the control (set at 100%, dotted line).

As previously mentioned, it is widely acknowledged that microorganisms involved in root canal infections exist not only as free-floating planktonic cells, but also as organized communities that adhere to the walls of the root canal, known as biofilms [64]. Sessile cells within biofilms exhibit significantly higher tolerance to most antimicrobial agents and the host’s defenses when compared to their planktonic counterparts [65]. Eliminating this population is crucial, as its persistence within the root canal system can result in the re-establishment of the endodontic infections [66]. Thus, the subsequent assessment addressed the antimicrobial impact of the BBR-free drug and BBR-loaded PLGA nanoparticles against biofilm formation of *E. faecalis* e *C. albicans*, in the concentration range already tested for the effect in the planktonic population. Simultaneously, the extended exposure duration, i.e., 24 h, provided insights into the influence of residual nanoparticles that might persist within the root canal structure post-irrigation. Results are summarized in Figure 5. Free BBR exhibited a dose-dependent anti-biofilm activity at 125 and 250 μg/mL on both pathogens. The sessile population of *E. faecalis* was also susceptible to the BBR-loaded PLGA nanoparticles, with inhibitory effects in significantly lower levels, namely in the 20 to 40 μg/mL BBR range. However, in these levels, BBR-loaded PLGA nanoparticles were not active against *C. albicans* biofilms, since the observed effect was similar to that found with the unloaded nanoparticles (corresponding to 285 to 570 μg/mL in the polymer). In fact, in the sessile population, the internalization of BBR-loaded nanoparticles is expected to be hindered within the biofilm structure. Thus, the levels of the released BBR might be insufficient to achieve effective intracellular inhibitory effects. In line with this reasoning, the efficacy of the loaded nanoparticles is expected to decrease in the sessile microbial population. Accordingly, results showed decreased efficacy against sessile *E. faecalis* compared to that observed with the planktonic population. This scenario is worsened in the *C. albicans* biofilm. In this condition, the BBR-loaded nanoparticles lack efficacy. This might be related to the features of the *C. albicans* biofilm [34], in which the yeast cells are protected and surrounded by extracellular polymeric substances, hindering the internalization of the nanoparticles so preventing intracellular BBR inhibitory mechanisms. As mentioned above, the non-internalized BBR nanoparticles would release low BBR levels to cause extracellular inhibition. Of note, empty nanoparticles also displayed some inhibitory activity, although with different meanings compared to the BBR-loaded nanoparticles. In the tested experimental conditions, the empty nanoparticles did not affect the planktonic population, and antimicrobial activity was only detectable in the sessile population. On *E. faecalis*, the empty particles had a small contribution on the effect observed with the loaded nanoparticles and, on *C. albicans*, they appeared to be only responsible for the observed antimicrobial effect. It should be noted that the tested nanoparticles bear a high polymer concentration, ranging from 285 to 570 μg/mL, which suggests non-specific surface-mediated inhibitory effects. In the same experimental setup, free BBR inhibited both planktonic and sessile populations, although at significantly higher levels, i.e., ≥125 μg/mL, compared to that accomplished by the BBR-loaded nanoparticles. The need of higher doses of free BBR might suggest the relevance of antibacterial surface-mediated mechanisms as observed before [63] which may be further supported by the low cellular availability of this molecule [67].

### 3.5. Cytocompatibility Assays

**Figure 6:**
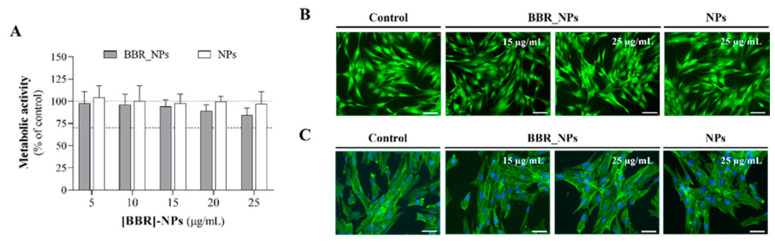
Human gingival fibroblasts’ response after exposure to BBR-loaded PLGA nanoparticles ([BBR]-NPs) and unloaded nanoparticles (NPs) regarding (**A**) metabolic activity, (**B**) live cells (green)/dead cells (red) assay and (**C**) cellular morphology after immunostaining for F-actin (green) and the nucleus (blue). (**A**) control was set at 100%; (**B**,**C**) scale bar of 50 µm.

During endodontic treatment there is a risk of apical extrusion, although rare. Studies have indicated that the occurrence of NaOCl extrusion is infrequent, with a rate of less than 1% [68]. In the event of such an occurrence, it could result in the movement of substances, particularly the irrigant, from the root canal into the periapical tissues. For instance, NaOCl, considered a standard irrigant, can lead to severe injuries if it is extruded into the periapical area. This is attributed to its strong oxidation and high alkaline characteristics that result in symptoms such as acute pain, swelling, ulceration, nerve impairment, and tissue necrosis, among other effects [69,70]. Considering this potential risk and its projected biomedical application, an assessment of the cytocompatibility of BBR-loaded PLGA nanoparticles was carried out using human gingival fibroblasts. Following the 1 h exposure period to the loaded nanoparticles, the viability and metabolic activity of the fibroblasts were not affected, as shown by the MTT reduction assay and live/dead assay (Figure 6A,B). The absence of toxicity was confirmed by the observation of the morphology, cell layer pattern and organization of the F-actin cytoskeleton in stained cultures (Figure 6C). Cells exhibited distinct nuclei and a flat, spindle-like morphology, with elongated F-actin filaments and maintained intimate cell-to-cell contact forming an organized layer. Within eukaryotic cells, the actin cytoskeleton is associated with the plasma membrane, being essential to cell structure/shape and several fundamental cellular processes such as division, polarization, endocytosis, and motility [71]. This structure is an initial target for cellular damage and toxicity, and cytoskeleton disruptions are among the most cytotoxic effects [72]. The immunostained cytoskeleton images (Figure 6C) are suggestive of an absence of cell toxicity induced by the BBR-loaded and unloaded particles. Regarding the empty nanoparticles, this result aligns with the known attributes of PLGA nanoparticles, which possess low cytotoxicity and good biocompatibility, rendering them an ideal carrier material [73,74].

Despite the absence of toxicity of the developed BBR-loaded PLGA nanoparticles, it should be emphasized that in the event of an extrusion, in an in vivo context, the irrigant will enter a dynamic and intricate tissue environment, where it will undergo rapid dilution, ultimately reaching concentrations below cytotoxic thresholds.

## 4. Conclusions

The results of the present work clearly demonstrated that free BBR exhibited dose-dependent inhibitory effects against both planktonic and sessile populations of *E. faecalis* and *C. albicans*, in relatively high levels, i.e., ≥125 μg/mL, compared to conventional antimicrobial drugs. Such high doses raise safety concerns with the possibility of adverse effects on the host. In this context, the BBR-loaded PLGA nanoparticles were developed to prevent adverse side effects on endodontic applications. This study reveals, for the first time, that PLGA nanoparticles as a delivery system for BBR exert potent antimicrobial activity towards planktonic endodontic pathogens, namely, *E. faecalis* and *C. albicans*. The findings show that the developed PLGA nanoparticles have an encapsulation rate of 77%, which is sufficient to produce inhibitory effects similar to those of its free form in significantly lower concentrations (about 10 times lower). This improvement could be attributed to the ability of the PLGA nanoparticles to enhance the water solubility and stability of drugs, enhance cellular diffusivity, and to prolong the release of BBR, resulting in more effective biological effects than the administration of free BRR as an irrigant. In addition, the BBR-loaded nanoparticles remained stable over 20 weeks of storage, indicating their suitability for clinical use during treatments. It is also verified that the developed nanoparticles are non-toxic for gingival fibroblasts at concentrations of 25 µg mL^−1^ in BBR, and that this concentration exerts antioxidant and antimicrobial activities. The use of nanoparticles provides an additional advantage in accessing the intricate structure of the root canal system, facilitated by the irrigation effect. Recognizing that an ideal endodontic irrigant should possess broad and long-lasting antimicrobial activity while demonstrating minimal cytotoxicity to periapical tissues and oral mucosa, the developed BBR-loaded nanoparticles represent a positive step towards meeting these criteria.

## Figures and Tables

**Table 1:** Characterization of unloaded and BBR-loaded PLGA nanoparticles.

Formulation	Size (nm)	PDI	ζ-Potential (mV)	EE (%)	DL (%)
PLGA	148 ± 2	0.26 ± 0.01	−6 ± 1		
BBR-loaded PLGA	140 ± 2	0.25 ± 0.01	−4 ± 1	67 ± 4	7 ± 1

Data represents mean ± standard deviation (n = 6). PDI—polydispersity index; EE—entrapment efficiency; and DL—drug loading.

## Data Availability

Data are contained within the article.

## Supplementary Materials

The following supporting information can be downloaded at: https://www.mdpi.com/article/10.3390/pharmaceutics16060786/s1. Figure S1. Characterization of the polymeric nanoparticles. (A) TEM images of PLGA and BBR-loaded PLGA nanoparticles. Scale 200 nm. FT-IR spectra (B) of raw BBR and PLGA powder and lyophilized nanoparticles PLGA and BBR-loaded PLGA. Table S1. Value of *r*^2^ obtained from the release data for different models of mechanism of drug release.
