# Supplementary material for: Improving the Antimicrobial Potency of Berberine for Endodontic Canal Irrigation Using Polymeric Nanoparticles
Source: Pharmaceutics. 2024 Jun 9;16(6):786. doi: 10.3390/pharmaceutics16060786 (PMC11207060; doi:10.3390/pharmaceutics16060786)
Supplement: Supplementary file 1 [file pharmaceutics-16-00786-s001.zip › pharmaceutics-2980660-supplementary.pdf]

Improving the Antimicrobial Potency of Berberine for Endodontic Canal Irrigation Using Polymeric Nanoparticles

Célia Marques[1,2], Liliana Grenho[3], Maria H Fernandes[3], Sofia A. Costa Lima[4]

SUPPLEMENTARY INFORMATION

Characterization of the polymeric nanoparticles

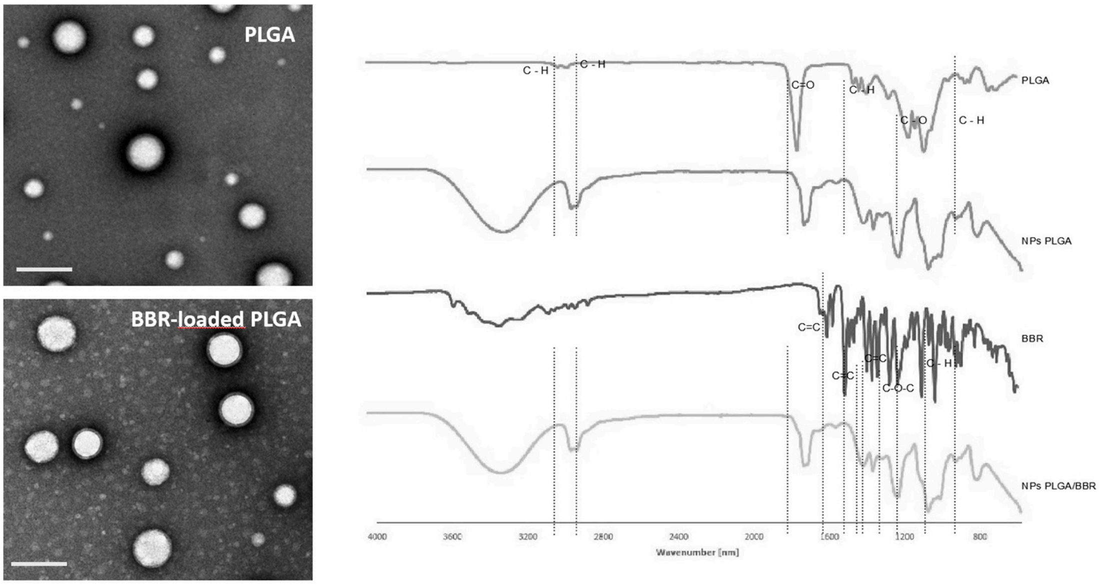

Figure S1. Characterization of the polymeric nanoparticles. (A) TEM images of PLGA and BBR-loaded PLGA nanoparticles. Scale 200 nm. FT-IR spectra (B) of raw BBR and PLGA powder and lyophilized nanoparticles PLGA and BBR-loaded PLGA.

Drug release kinetics

The following mathematical models for drug release kinetics were applied to evaluate the mechanism of drug release:

<u>Zero order release model</u>, refers to the process of constant drug release from a drug delivery device

Q = Q0 + K0t

Q: amount of drug released or dissolved; Q0: initial amount of drug in solution (it is usually zero); K0 zero order release constant.

First order release model, drug release rate depends on its concentration
Log C = Log C0 - kt / 2.303
C0: initial concentration of drug; K: first order constant.

Hixson-Crowell release model, describes the release from systems where there is a change in surface area and diameter of particles.
$Q_0^{1/3} – Q_t^{1/3} = K_{HC}$ t
Q_t: amount of drug released in time t; Q_0: initial amount of the drug in tablet/ formulation; $K_{HC:}$ rate constant for Hixson-Crowell rate equation.

Higuchi release model, relate the drug release rate to the physical constants based on simple laws of diffusion
$Q_t = k_H (t)^{0.5}$
Qt: amount of drug released in time t; T: time in hours; kH: release rate constant for the Higuchi model

Korsmeyer-Peppas release model,
F=Mt/M N = Ktn
F: fraction of drug release at time t; Mt / M: fraction of drug released at time t, ; K: the rate constant
n: release exponent.

The model that best fits the experimental release data will be selected based on the correlation coefficient (R^2).

Table S1. Value of r^2 obtained from the release data for different models of mechanism of drug release.

Model	BBR-PLGA nanoparticles	Free BBR
**Zero-order**	0.8713	0.8831
**First-order**	0.8920	0.9765
**Higuchi**	0.9906	0.9471
**Hixson-Crowell**	0.9640	0.9047
**Korsmeyer-Peppas**	0.9810	0.9162

The BBR release from the nanoparticles, under the studied conditions, was diffusion controlled described by the Higuchi model, as plots of the amount released versus square root of time was found to be linear. When the log % of drug remaining to be release versus time was plotted, in accordance o first order model, curves were obtained (r^2<0.8920) indicating that drug release do not follow first order. BBR released do not depend on its concentration, but on its diffusion throughout the polymeric matrix.
